# Multi-defect detection and classification for aluminum alloys with enhanced YOLOv8

**DOI:** 10.1371/journal.pone.0316817

**Published:** 2025-03-20

**Authors:** Ying Han, Xingkun Li, Gongxiang Cui, Jie Song, Fengyu Zhou, Yugang Wang

**Affiliations:** 1 Naval Architecture and Port Engineering College, Shandong Jiaotong University Weihai, Weihai, Shandong, People’s Republic of China; 2 School of Control Science and Engineering, Shandong University, Jinan, Shandong, People’s Republic of China; Semnan University, IRAN, ISLAMIC REPUBLIC OF

## Abstract

With the increasing application of aluminum alloys in the industrial field, the defect of aluminum alloys significantly impacts the structural integrity and safety of products. However, state-of-the-art material defect detection methods have low detection accuracy and inaccurate defect target frame problems. Therefore, an enhanced YOLOv8-ALGP (aluminum, Ghost, P2) defect detection and classification method for 13 defects is proposed in this paper. Firstly, based on the AliCloud Tianchi dataset, 3 defects are added and an enhancement strategy is implemented to increase the diversity of the training dataset, which improves the generalization ability of the model. Secondly, an ALGC3 (aluminum, Ghost, Concentrated-Comprehensive Convolution Block (C3)) module is introduced to optimize the fusion of Ghost convolution and residual connectivity, resulting in a more lightweight model. Finally, the backbone network structure is reconstructed. Fine-grained adjustments and improvements are made to enhance neck network layers and the feature extraction capability. Defect features are extracted and analyzed more efficiently, and the network model better identifies defects such as jet, camouflage, etc. The average detection rate of all defects in the data set is improved. As a result, the average detection rate of all defects in the dataset is improved. Experimental results show that the proposed method performs effectively in target detection and classification. The number of model parameters is reduced from more than 300,000 to 160,000, significantly reducing the complexity of the model. In addition, the average detection accuracy is improved from 64.5% to 71.3% compared to the YOLOv8. In addition, the detection accuracies of effacement and jet defects, particularly, are improved from 21.6% and 38.5% to 32.2% and 60%, respectively. It shows that the proposed method can effectively identify the surface defects of aluminum alloys, which improves product performance in the aluminum industry.

## Introduction

Aluminum alloys play a significant role in industrial manufacturing [1]. They have become essential materials in industrial manufacturing and are widely applied in many countries. In industrial manufacturing, aluminum alloys may have defects such as scratches, orange peel, pits, and dirty spots [2]. Incalculable consequences will arise if these defects are ignored and not detected and processed promptly [3]. Therefore, accurately detecting and classifying defects in aluminum alloys are becoming increasingly significant.

Artificial defect detection is easily affected by subjective factors, which do not meet the requirements for real-time detection, and is gradually being replaced by traditional methods [4–6]. Subsequently, a series of defect detection methods has been proposed by researchers. Shaloo et al. [7] stated that laser-induced breakdown spectroscopy is capable of detecting defects such as porosity by evaluating the chemical elements of the sample, while the other types of defects in fusion welding were ignored. Parlak [8] presented YOLOv5 to detect internal defects on X-ray images of high-pressure aluminum die castings, achieving high precision and fast processing. However, X-rays might cause harm to researchers, and this detection method was relatively rare in general examinations. The Norwegian company Elkem [9–11] developed the first infrared detection equipment, Therm-O-Matic, for an automatic detection system of surface defects in continuous casting steel billets. This equipment commonly detects surface defects in aluminum sheets and components.

With the development of intelligent algorithms, traditional detection methods could not meet the demand due to the low precision and limited detection types [12,13]. Deep learning techniques have attracted researchers’ attention, and state-of-the-art achievements in defect detection have emerged [14–17] . Li et al. [18] proposed a lightweight detection model for M2-BL-YOLOv4. However, the defect types and dataset are limited, thus limiting its generalization ability. Li et al. [19] improved the faster region-based convolutional neural network (R-CNN) algorithm, combined with data augmentation, ResNet50, and a path-enhanced feature pyramid network, to enhance the accuracy of surface defect detection in industrial aluminum products. Chen et al. [20] proposed an enhanced model called RER based on YOLOv5, which improved the model’s ability to extract rich features accurately. Yan et al. [21] developed a composite vision system that simultaneously performs three-dimensional depth and two-dimensional grayscale imaging to detect typical surface defects in aluminum alloys. Xu et al. [22] proposed a BHE-YOLO small target detector, which outperforms traditional algorithms in various indicators, especially in detecting minor target defects. However, the algorithm’s generalization ability might vary under different types and environmental conditions. Wei et al. [23] used deep learning networks to detect surface defects on aluminum profiles, demonstrating the effectiveness of the network in detecting surface defects on aluminum profiles. Hai et al. proposed a deep learning-based method that effectively improves defect detection performance, especially in identifying minor porosity defects, by combining the gain adaptive multi-scale Retinex algorithm with the feature pyramid network and the convolutional block attention module [24]. However, they focused on studying the structure of the algorithm while neglecting to improve the dataset. In contrast, Matin M. et al. [25] presented the K-means algorithm to estimate the transition fatigue life of piston aluminum alloys. The researchers fitted the algorithm using an experimental dataset and employed preprocessing methods and kernel functions to improve performance. While different in approach, both studies work to improve the accuracy and efficiency of materials performance evaluation, demonstrating the wide range of applications and potential of machine learning in materials science.

To improve the detection accuracy, Xu et al. [26] proposed a GAE-cascade R-CNN model that effectively improved surface defect detection accuracy and average precision in aluminum profiles through deformable convolution, guided anchoring, and sample enhancement techniques. Shen et al. [27] proposed a defect detection model based on multi-task deep learning, which improved the accuracy and segmentation performance of aluminum defect detection on limited datasets by adaptively balancing multi-task loss layers with weights. At the same time, it performed defect detection, multi-label classification, and area segmentation, improving efficiency. Zhou et al. [28] proposed the YOLOv8-EL detection method, which employs data augmentation to address the issue of imbalanced datasets. They also integrated CAM into the backbone network, improved the feature extraction network, and further enhanced detection accuracy with the multi-attention detection head. This method improves feature utilization and achieves a detection accuracy of 89.90% on the ELPV dataset. Lu et al. [29] proposed a strip surface defect detection algorithm called DCN-YOLO, which integrates lightweight convolution blocks of DSConv and an efficient channel attention module to create simplified models without compromising detection accuracy. Influenced by this development, incorporating lightweight networks to enhance the experimental results has been considered. The above research indicates that the YOLO algorithm performed well in aluminum alloys defect detection, attaining high accuracy and robustness [30]. However, it still faces challenges, such as limited generalization ability with small sample data and restricted recognition capacity for various defect types [31]. Deep learning research in this field has made progress, yet several challenges remain to be addressed.

Considering previous research, this article aims to enhance the size and quality of the aluminum alloys defect dataset, further optimize the YOLOv8 model structure, and create a more accurate and reliable system of aluminum alloys defect detection by tackling its shortcomings. Specifically, three types of defects are added, bruise, camouflage, and coating cracking. The type and number of defects in the dataset are enriched, and mosaic data enhancement can be used to simulate noise and interference in the image, thus improving the robustness of the model to noisy data. Then, a defect detection and classification method called YOLOv8-ALGP(aluminum, Ghost, P2) is proposed in this paper. Building upon the YOLOv8 network model, this approach introduces a lightweight fusion of the Ghost module and a P2 small target detection network model to enhance the model’s performance in detecting small defect targets while maintaining high computational efficiency. In addition, the backbone network of the model is redesigned. Strengthening the neck network layer makes the model more stable and improves the performance of the model for tasks such as classification and detection. The experimental results show that the method is effective in defect detection and classification.

The rest of this paper is organized as follows: The dataset, preprocessing and methods are described in Section sec2. The model training is described in Section sec3. Experiments are presented in detail in Section sec4, and the results demonstrate the effectiveness of the proposed methods. Conclusions are provided in Section sec5.

## Dataset and methods

### Dataset

In this paper, the publicly available dataset—AliCloud Tianchi dataset is selected [32]. The dataset contains more than 3,233 monitoring image data of aluminum profiles containing defects from actual production, each image contains one or more defects, and the resolution of the defect images are all 2560 × 1920.

**Fig 1:**
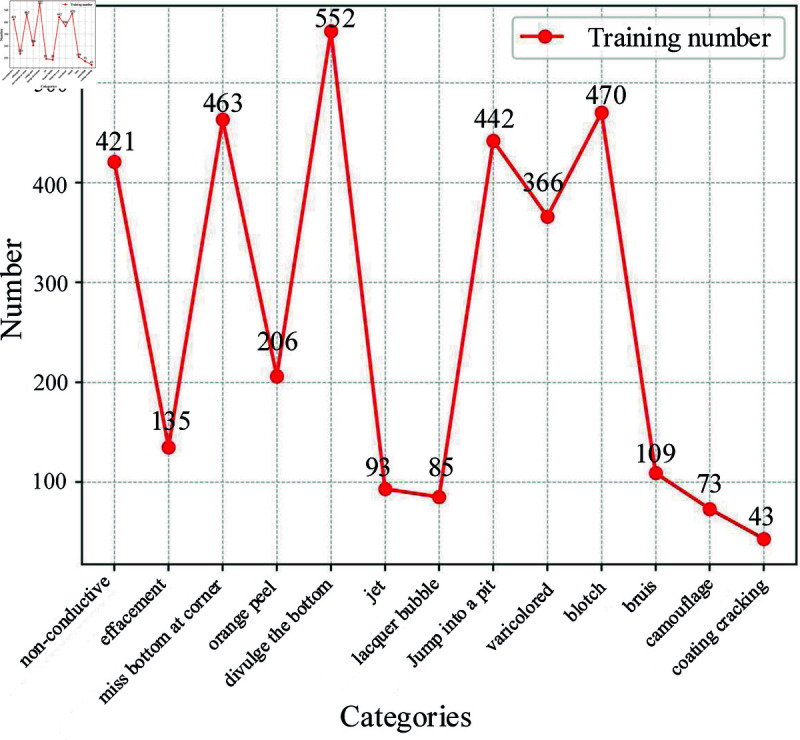
The number of defective images in the dataset.

The defect dataset is classified into ten major categories, which are shown in Fig 1. From the figure, there are fewer samples of camouflage, and coating cracking defects. Therefore, traditional data enhancement methods are used to solve the uneven distribution of certain defects, which is described in detail in the next section. After enhancement by these operations, the defect dataset contains a total of 3600 images, allowing for a balanced distribution of samples in the dataset, which is essential for improving the overall performance and usefulness of the model.

To further enrich our dataset and improve the generalization ability of the model, three new types of defects that are commonly found in aluminum alloys are added, bruise, camouflage, and coating cracking. Each type of defect has its unique features and details, and more data can help the model learn defect features better, thus improving the accuracy of the model in recognizing defects. By introducing these defect samples, the diversity of the dataset is improved so that the model can learn more kinds of defect features.

### Data preprocessing

The dataset should have a series of processes, especially for the adopted data enhancement algorithm, to improve the performance and training efficiency of the proposed model [33]. The preprocessing steps include 4 steps.

**Fig 2:**
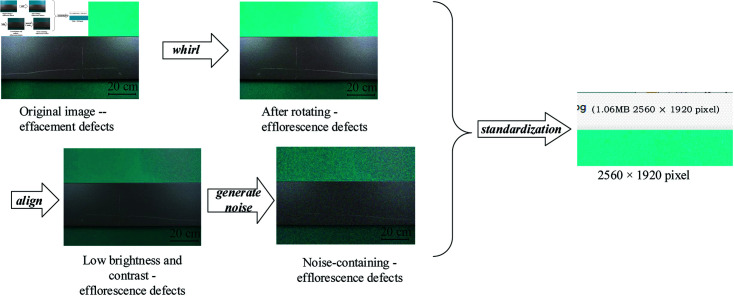
Flow of data enhancement method.

(1) Data cleaning. Invalid or incorrect image data and images with incomplete or incorrect label information should be deleted.

(2) Data enhancement. As shown in Fig 2, the image is randomly transformed in several ways. These operations do not change the category of images however increase the number of training samples and generate more training samples, thus improving the generalization ability and effect of the model.

**Fig 3:**
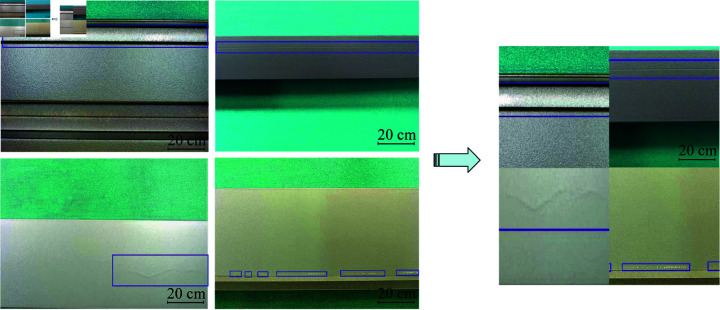
Mosaic data enhancement method.

In the course of exploring methods to effectively expand the dataset and enhance model training efficacy, we note the significant findings by Parlak I.E. and Emel E. They significantly increased the size of the dataset by implementing data augmentation techniques such as rotation, sharpening, and translation. After these treatments, the total number of images in the dataset increased to 3,466 [8]. Similarly, researchers such as Chen K. et al. expanded the dataset by adopting an image flipping strategy, which increased the capacity of the original training dataset by four times and optimized the training effect [34]. In this study, we employed the mosaic data augmentation algorithm to enhance the diversity of the dataset by randomly cropping four images and merging them into a composite image. Each picture has a corresponding target box. After splicing, the corresponding box of the picture is obtained. Passing such a new image into the network as training data not only greatly enriches the background of detecting object but also computes the data of four images at once during the batch normalization calculations, as shown in Fig 3.

**Fig 4:**
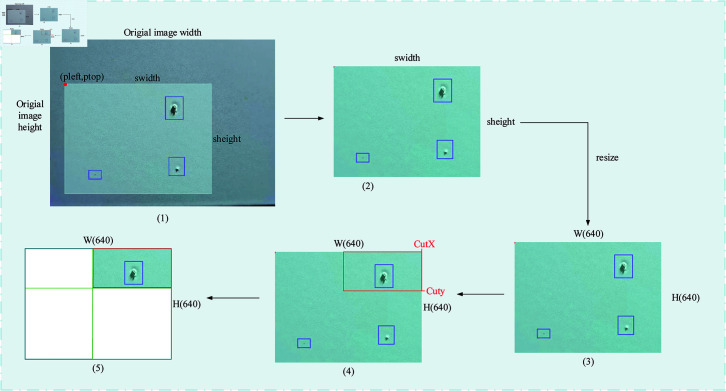
Structure of Mosaic data enhancement method.

The Mosaic data augmentation method is divided into four steps. First, four images from the dataset are randomly selected. Then, flipping, scaling and color gamut changes are performed. Moreover, the original images are sequentially arranged in the top left, bottom left, bottom right, and top right positions. Finally, the selected area of the four images is cut down by the matrix method, and a new picture is stitched together, including a series of contents such as the defect check box. The entire mosaic process is shown in Fig 4.

(3) Image unification. The pixel value is uniformly set to 2560 × 1920 in this paper, reducing the impact of size differences between different images on model training.

(4) Dataset partitioning. The dataset, divided into training, validation, and testing sets, helps evaluate the generalization performance of the model, adjust the hyperparameters during the training process, select the best model structure. Table 1 shows the proportional division of the data and the number of images in the final dataset.

**Table 1:** Dataset splitting.

Dataset	partition suggested proportion	actual partition proportion
**Training set**	70–80%	70.01%
**Validation set**	10–20%	10.02%
**Test set**	10–20%	19.91%

Data preprocessing is essential to ensure the quality of the dataset and to improve the training efficiency and final performance of the model. Uncertainty in the training process can be reduced and the accuracy and reliability of the model can be improved by proper preprocessing. In this regard, Matin M et al. [35] conducted a study on the effect of manufacturing and environmental parameters on the arc pressure of medium voltage switchgear and found that the initial pressure, the width of the main and the condition of the pipeline are the key factors that affect the pressure increase. This finding highlights the importance of careful preprocessing of data prior to model training. Similarly, we clearly demonstrate the different values of losses in section sec3, as shown in Fig 7, by studying the learning curves in the professor’s article. As can be seen in Fig 7(b) and 7(e), the classification loss for training and validation starts to decrease rapidly and gradually stabilizes, indicating that the performance of the model is stabilizing. This process once again demonstrates the key role of data preprocessing in improving the efficiency and performance of model training.

### Basic structure of the proposed approach

**Fig 5:**
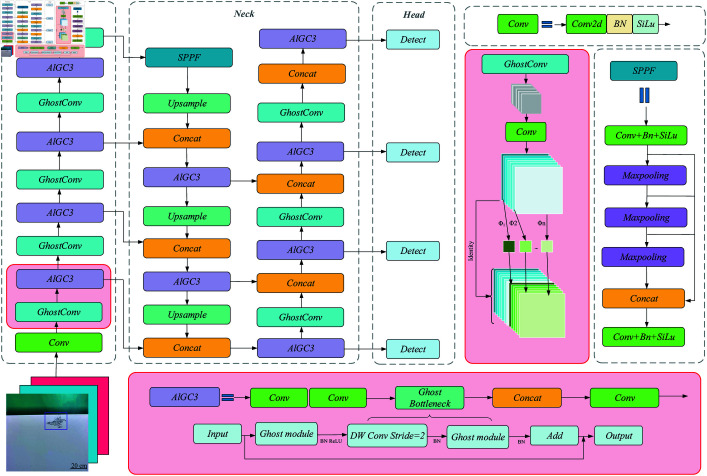
YOLOv8-ALGP network architecture.

The YOLOv8 model is adopted and developed to achieve fast and accurate object detection and thus solve the issues described in the introduction. The new overall network structure of the method is shown in Fig 5.

Fig 5 shows that the YOLOv8-ALGP network consists of a backbone, neck, and head. According to the defect dataset, aluminum alloys defects are irregular. Therefore, Ghost and P2 small target detection structure is integrated into YOLOv8, which improves the detection efficiency and classification accuracy of aluminum alloys defects.

- Aluminum alloys defect feature extraction

**Fig 6:**
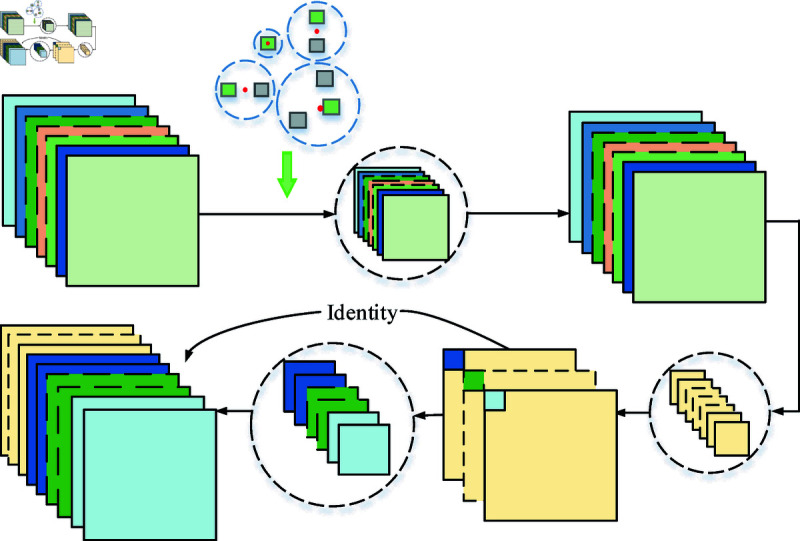
Architecture diagram of DSC-GhostConv.

The Convolutional Neural Network (CNN) part of YOLOv8 is not ideal for feature extraction of aluminum alloys defects. Therefore, the backbone part is improved on this basis. The backbone network Ghost convolution (GhostConv) module of YOLOv8-ALGP replaces the Convolutional module of the v8 algorithm. The C3 structure and the Ghost module form a new structure, ALGC3(aluminum, Ghost, Concentrated-Comprehensive Convolution Block(C3)). ALGC3 replaces the CSP Bottleneck with 2 convolutions (C2f) modules of the v8 algorithm. Combining GhostConv with Deep Separable Convolution (DSC) yields a new convolutional block, DSC-GhostConv, which combines the advantages of DSC and reduces the total computational effort and complexity of the model. The principle of DSC-GhostConv is shown in Fig 6.

Fig 6 shows that using DSC to replace the original standard convolution of Ghost can significantly reduce the number of network parameters, FLOPs, and the cost of resources without changing the size of the output feature map. The size of the output feature map is *f* × *f* , the number of input channels is *i* , and the output channel is *o* . The formula for calculating the number of parameters of DSC-GhostConv is as follows [36],


$$\begin{array}{ccc} k\times k\times i+i\times o & & \end{array}$$ (1)

The calculation formula of DSC-GhostConv is as follows [36],


$$\begin{array}{ccc} k\times k\times i\times f\times f+i\times o\times f\times f & & \end{array}$$ (2)

- Network structure algorithm

ALGC3 replaces the original C2f module of YOLOv8 and makes up for the deficiencies of the original C3 module. In this way, the two paths can be better combined to reduce the model complexity without affecting the model performance. The network structure algorithm is shown in Table 2 below.

**Table 2:** Network architecture algorithm.

Algorithm 1 Network Architecture
**Input:** *in_channels*, *out_channels*, *expansion*=0.5, *depth*=3
**Output:** The *out* through the residual connection and the final ReLU activation.
① *hidden_channels* = int(*out_channels*×*expansion*)
② self.ghost1 = GhostModule(*in_channels*, *hidden_channels*)
③ self.ghost2 = GhostModule(*hidden_channels*, *out_channels*)
④ self.layers = nn.ModuleList([...])
⑤ *x* = self.ghost1(*x*)
⑥ *y* = self.layers0
⑦ for *layer* in self.layers[1:]: *y*=*y*+layer(*x*); *y*=F.relu(*y*)
⑧ *out* = self.ghost2(*y*)
⑨ *out*=*x*+*out*; *out*=F.relu(*out*)
**Return *out*. **

The algorithm is detailed in Table 2, which uses the input and output channels as the corresponding results through the residual connections and the ReLU activation function. Firstly, number of hidden channels are calculated. Then, two Ghost modules and a layer of C3 structure are created. The input passes through them with each layer having residual connections and ReLU activation. Finally, the final output is passed through the second Ghost module with residual connections and final ReLU activation.

- Introduction of P2 small target detection

Introducing P2 small target detection into the algorithm structure strengthens the performance and robustness of the algorithm. It extracts critical information and features related to the target from the defects, which is significant in target identification and tracking tasks such as automatic driving, security monitoring, and other fields. Introducing the algorithm system can locate and identify defects more accurately and reduce the misjudgment rate, thus improving the overall performance of the aluminum alloys inspection system.

The last part of the network structure is the classification and regression layer, in which the softmax activation function is introduced to convert the neural network output into the probability distribution of the class. The category identification probability of each defect is between 0 and 1. The sum of the 13 defect probabilities is 1 to predict the category of aluminum alloys defects and the specific location information of defects. The defects are marked with a boundary box.

## Model training

**Fig 7:**
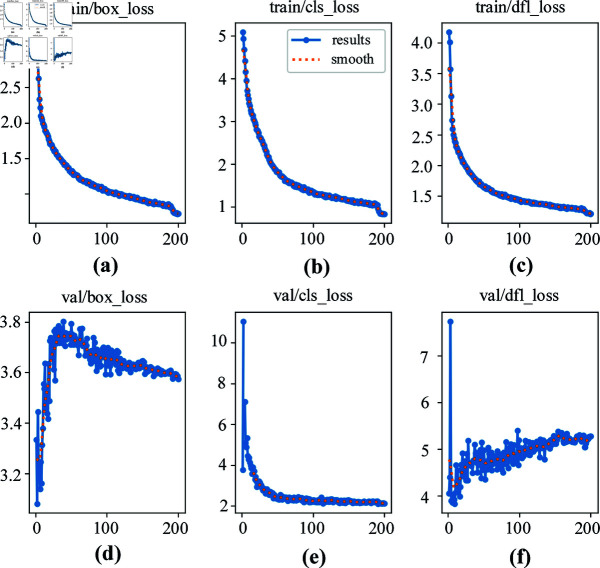
Various types of loss curve charts.

Certain losses occur in the network model training for defect detection, so outputting all the losses is necessary. Observing the loss value graph to judge the detection efficiency of the network model not only helps evaluate the performance of the model but also guides the adjustment of network parameters to optimize the performance. In this training, various losses are included, such as train/box_loss, train/cls_loss, train/dfl_loss, val/box_loss, val/cls_loss, and val/dfl_loss. The loss curve in the training process is shown in Fig 7, in which various network losses are recorded once each round.

Fig 7 indicates that the boundary box regression loss and classification loss value show a downward trend. At the beginning of the training, the loss value decreases significantly, indicating that the gradient descent will be carried out only when the learning rate is selected correctly.

The above improvements and innovations in the backbone network, neck, and other aspects reduces the computational complexity of YOLOv8-ALGP while maintaining high performance and improving the accuracy and speed of classification and detection of aluminum alloys defects.

## Experiment results and discussion

**Fig 8:**
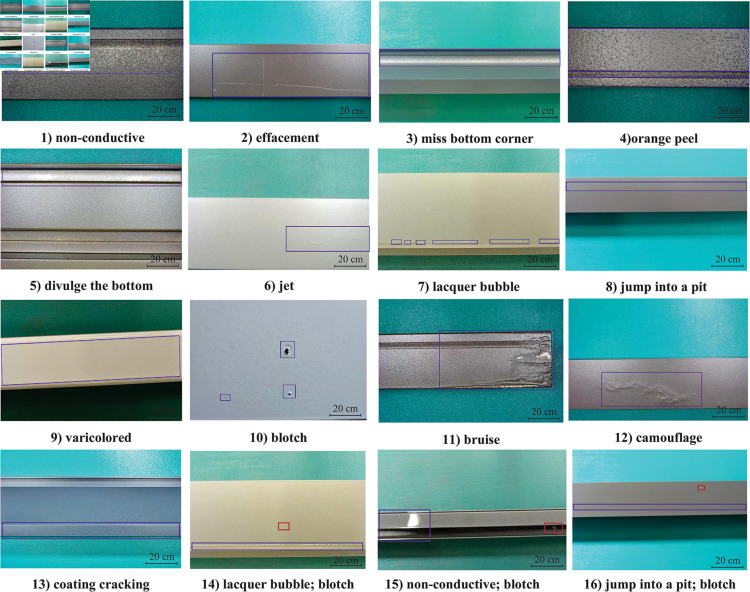
Example diagrams of various defects.

The experiment defect dataset is described in detail in section ach image contains at least one defect, which is shown in Fig 8. Defects are labeled using the Labeling tool, which creates rectangular boxes to delineate the defect areas. In addition, the labeled information is saved as an XML file in PASCAL VOC format for subsequent model training.

### Experimental environment

All experiments were edited in Visual Studio Code under the Ubuntu 22.04.4 operating system, connected to a remote server using NVIDIA GeForce RTX 3060 GPU with 12 GB memory and Python 3.9.0 programming language. Deep learning libraries such as TensorFlow and PyTorch were imported, CUDA12.2 and CUDNN 8.9.2 were used to accelerate GPU, and training was performed based on the PyTorch 2.3.1 deep learning framework.

Before the training, the size of the defect images was uniformly processed. The resolution was scaled from the original 2560 × 1920 to 640 × 640, the training period (epochs) was set to 200 rounds, and the number of images in each batch (batch size) was set to 32. The GPU training was specified by default, the number of workers was set to 8, and the optimization algorithm was Stochastic Gradient Descent (SGD). The learning rate was 0.01, and the weight decay was 0.0005. The mean Average Precision (mAP) measures the model in the final test.

### Experimental evaluation indicators

In this paper, experimental indicators are mainly observed to evaluate the model’s detection effect accurately. The experimental indicators include confusion matrix, recall rate, Precision-Recall curve (P-R curve), balanced F Score curve (F1 score curve), and accuracy rate. The confusion matrix is shown in Table 3.

**Table 3:** Confusion matrix.

**True Class**		**True Value**	
		Positives	Negatives
**Predicted Value**	Positives	TP	FP
	Negatives	FN	TN

From Table 3, true positive denotes the predicted correct target type, true negative presents the correctly identified negative sample that is a negative sample, false negative means the missed target that should be detected, and false positive denotes the wrongly identified negative sample as a positive sample.

**Fig 9:**
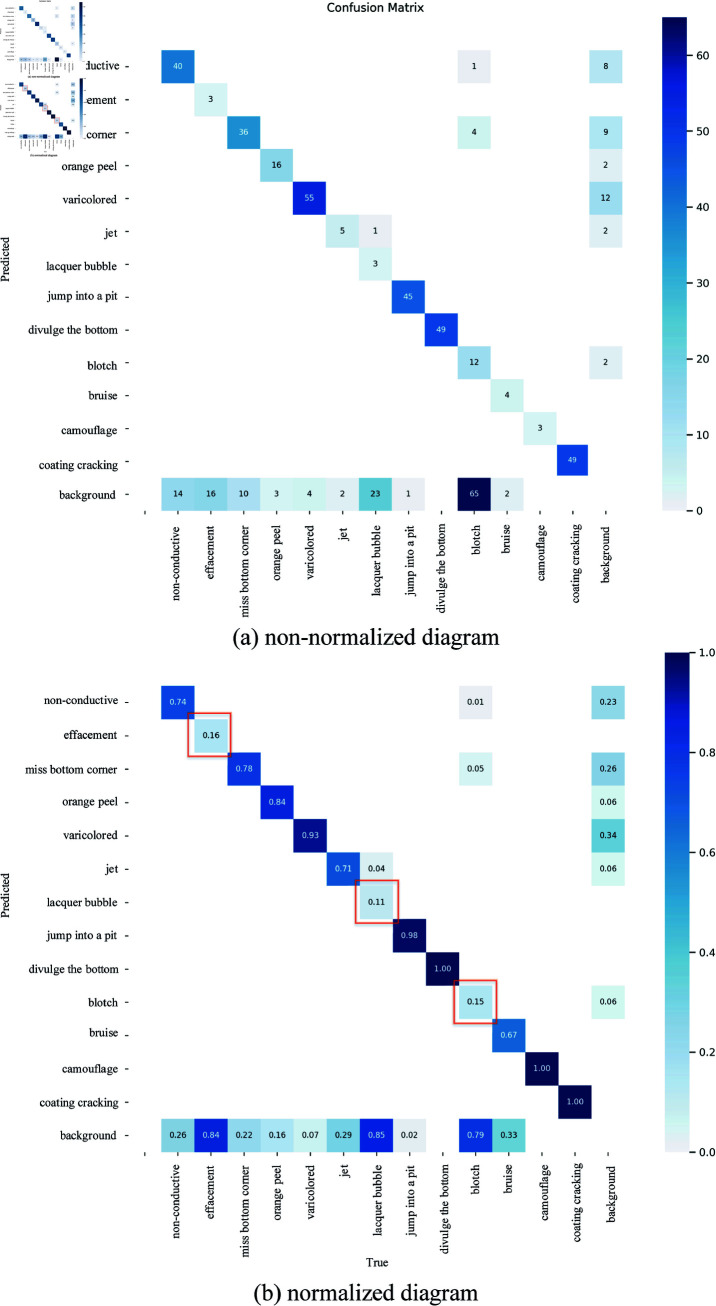
Confusion matrix diagram.

Fig 9(a) shows the image without normalization. It may have the shortcomings of contrast problem and increased computational complexity. As shown in Fig 9(b), the value on the diagonal indicates the proportion of correct prediction, and a high value corresponds to a high number of correct predictions. The correct prediction rate of the three defects of scratch, paint bubble, and dirty spot is low because of the small defect proportion area. In addition, the defects of scratch and paint bubbles are linear, very small, or slender, and background noise is present on the surface of aluminum alloys. This defect can be confused with noise and can be challenging to identify. Therefore, this paper will focus on how to improve the recognition and classification accuracy of these defects.

**Fig 10:**
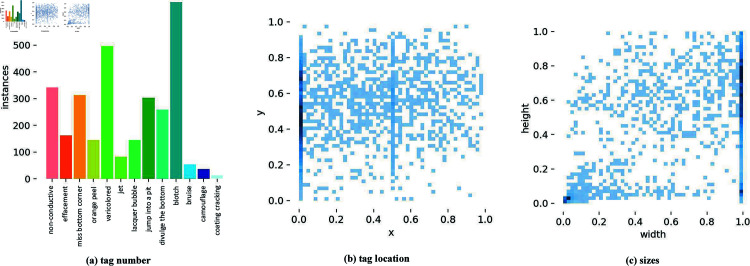
Tag distributions.

The number and distribution of objects in the training set are shown in Fig 10. Fig 10a shows the object names and corresponding quantities, clarifying how many images are present for each defect in the dataset. Fig 10b shows the distribution of labels. The X-axis is the ratio of the label center to the width of the image, and the Y-axis is the ratio of the label center to the height of the image. The figure shows distributions in all parts of the image, most of which are in the left middle of the image. Fig 10c shows the size of the object, with the X-axis representing the ratio of label width to image height and the Y-axis representing the ratio of label height to image height.

The precision is defined in Eq (3), which denotes the actual positive sample proportion in the identified positive samples [37].


$$\begin{array}{ccc} Precision=\frac{TP}{TP+FP}. & & \end{array}$$ (3)

The recall is expressed in Eq (4), which indicates the correctly identified proportion of positive samples [37].


$$\begin{array}{ccc} Recall=\frac{TP}{TP+FN}. & & \end{array}$$ (4)

The number and distribution of objects in the training set are shown in the Precision-Recall index, which shows the relationship between the accuracy and recall of the model under different classification thresholds.

**Fig 11:**
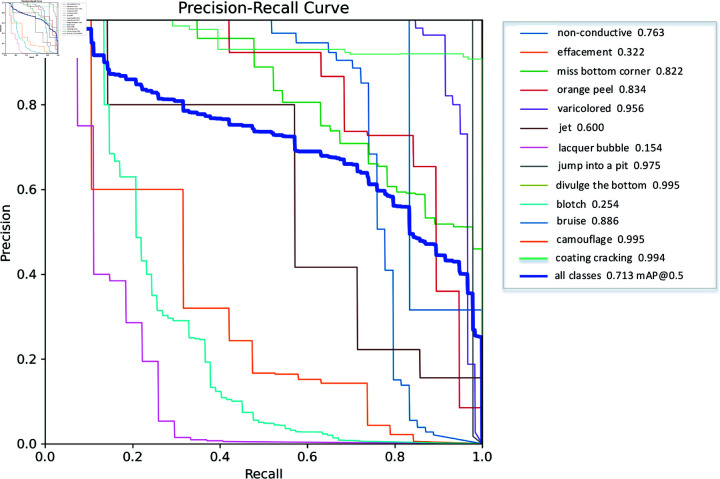
P_Rcurve diagram.

From Fig 11, when the curve is close to the upper right of the coordinate axis, it indicates better performance of the model and a high P-value for correctly classifying positive samples. However, when the curve is close the right, the recognition ability of the model for positive samples is poor, that is, its recall ability is poor.

**Fig 12:**
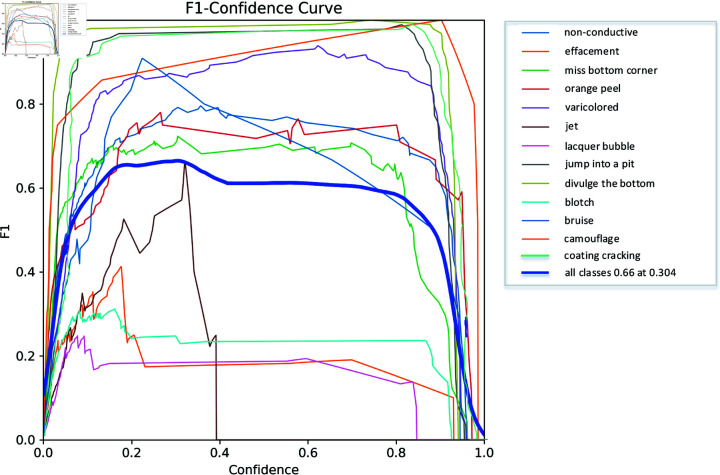
F1 score curve diagram.

As shown in Fig 12, the F1 score is the harmonic average of precision and recall, with higher values indicating more robust models. Its calculation formula is as follows [38],


$$\begin{array}{ccc} F1=\frac{2\times precision\times recall}{precision+recall}. & & \end{array}$$ (5)

### Experimental results

After completing the training of the network, we evaluated the multi-defect detection network using randomly selected test images from 13 defect categories in the entire dataset. The Average Precision (AP) and mAP scores were recorded for each of the 13 defect types. Subsequently, the results of the aluminum defect detection from the network are presented.

More specific data are listed for the two methods. The average detection accuracy of all defects increased to 71.3%. In particular, the detection accuracy of jet defects nearly doubled, and empirical methods are provided to improve other types of defects. The specific detection results of 13 kinds of defects are shown in Table 4.

**Table 4:** MAP Comparison of wo algorithms with different defects.

Class	YOLOv8 [42] (%)	YOLOv8-ALGP(%)
**all**	**64.5**	**71.3**
**non-conductive**	71.5	76.3
**effacement**	**21.6**	**32.2**
**miss bottom corner**	83.1	82.2
**orange peel**	85.7	83.4
**varicolored**	83.8	95.6
**jet **	**38.5**	**60**
**lacquer bubble**	36.5	15.4
**jump into a pit**	96.7	97.5
**divulge the bottom**	99.5	99.5
**blotch**	27.7	25.4
**bruise**	**69.3**	**88.6**
**camouflage**	99.4	99.5
**coating cracking**	99.5	100

**Fig 13:**
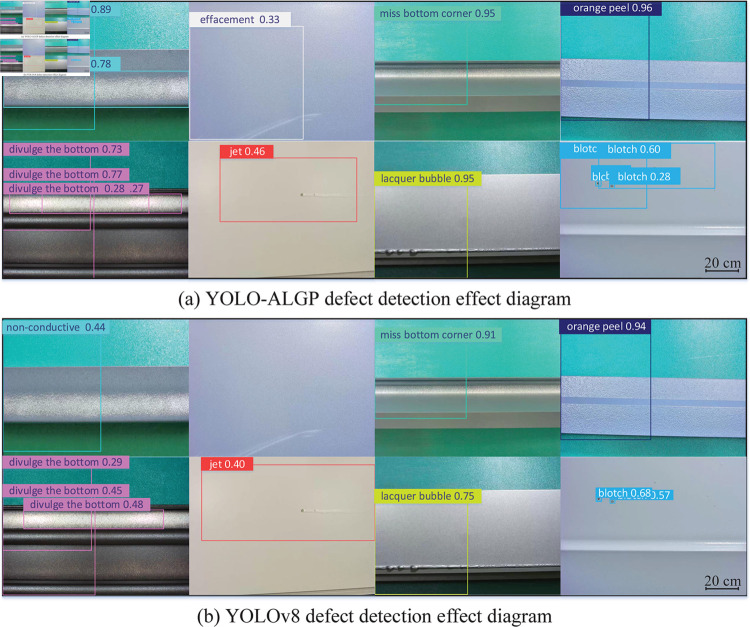
Partial defect detection and classification comparison diagram.

Some results of the defect detection are shown in Fig 13, which shows that the improved algorithm detects the correct rate greatly. Besides, the corrosion defects of the coating were detected successfully by the improved method. In summary, it shows that the YOLO-ALGP algorithm greatly improves the correct detection rate of defect.

**Fig 14:**
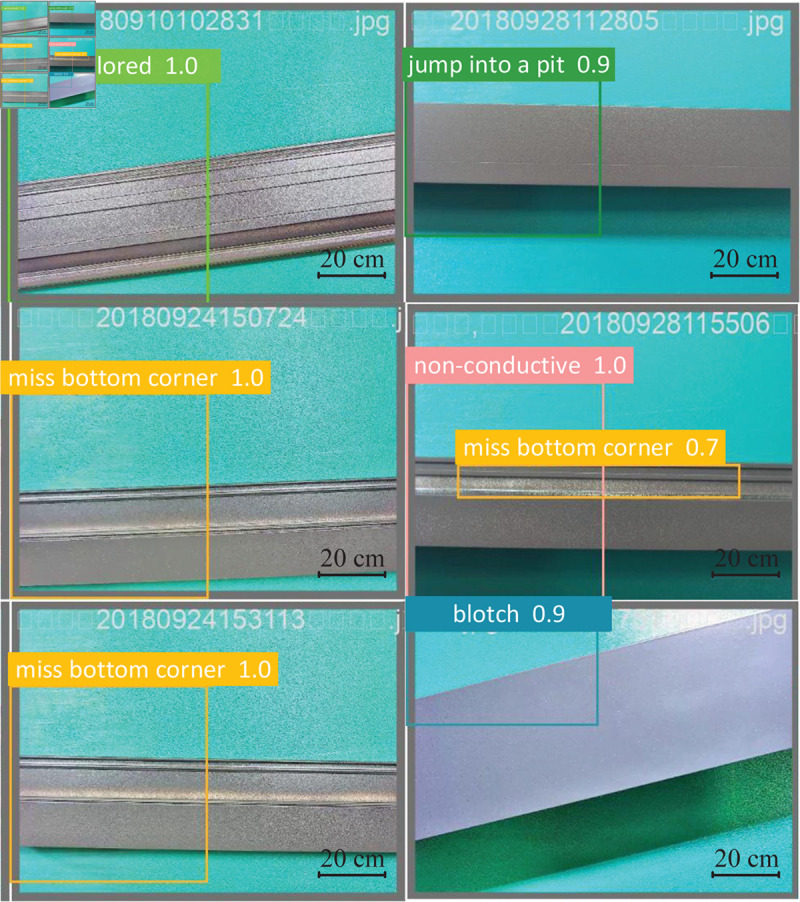
Defective training results.

Both the confidence score and the category prediction output a final score, indicating the probability that this bounding box contains an object of the defect type, usually ranging from 0.0 to 1.0, as shown in Fig 14. 1.0 indicates that the model is confident that the detection result is correct, and 0.7 close to 1 indicates that the model is moderately confident. In practical applications, the result with high confidence is usually taken as the final test result. In the figure, the detection of the two defects on the left is basically accurate, while the three defect categories on the right need to be verified.

**Fig 15:**
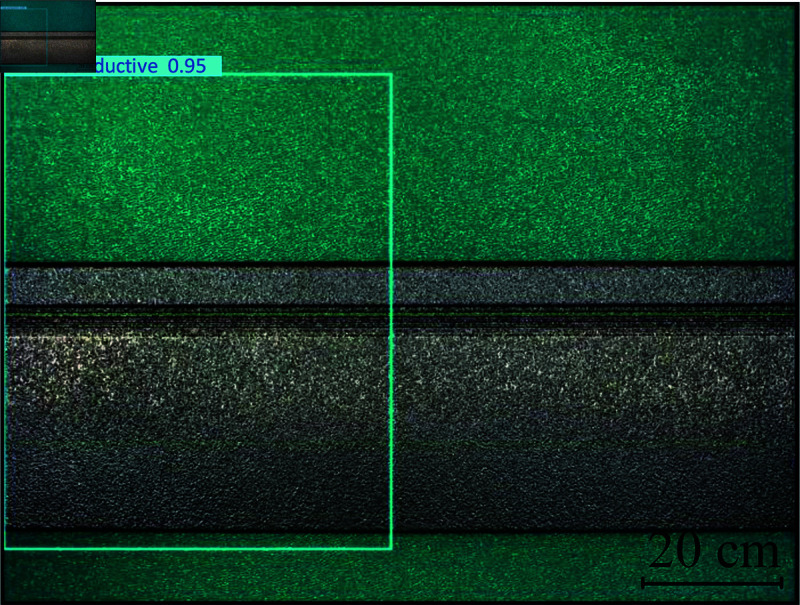
Non-conductive defect prediction.

The trained network model is saved, and a best-weight file is generated, which is used to detect the test graph. The model includes 380 layers, 1,602,628 parameters, 0 gradient, 8.6 GFLOPs, and the image pixel value for the test is 480 × 640. The preprocessing speed, inference speed, and post-processing speed are 4.3, 155.6, and 25.8 ms, respectively. The final prediction probability is 95%, and the defect is determined to be non-conductive. The prediction results are shown in Fig 15.

### Experimental discussion

The experimental results show that the improved YOLOv8 method can achieve an increase in the defect detection rate of aluminum alloys by introducing the Ghost module and the P2 small target detection network model. By applying the ALGC3 module, the complexity of the model is successfully reduced while the detection accuracy is enhanced. This improvement is well demonstrated in the average detection results for 13 different types of defects. In this study, the performance of the improved YOLOv8-ALGP algorithm is compared with the conventional YOLOv8 algorithm in terms of defect recognition accuracy. The comparative analysis shows that the YOLOv8-ALGP algorithm demonstrates higher accuracy in defect recognition and is able to detect almost all types of defects, thus validating the effectiveness of the improved algorithm. In summary, the results of the defect detection study clearly indicate that YOLOv8-ALGP has a significant advantage in terms of accuracy compared to other algorithms.

In order to further validate the effectiveness of YOLOv8-ALGP, its performance was compared with 10 other algorithms, including YOLOv5, YOLOv5-P2BiP, YOLOv6, YOLOv7, and YOLOv8n, in the detection of 13 types of aluminum alloys defects. The experimental results are presented in Table 5. Table 5 gives the values of different indicators in detail, where mAP represents the average accuracy across 13 defect categories, and mAP50 is the mAP value under the 50% Intersection-over-Union (IoU) threshold. mAP50-95 calculates mAP values in the 50%–95% IoU thresholds to more accurately evaluate the model performance under different IoU thresholds.

**Table 5:** Performance comparison of various algorithms.

Network	Precision (%)	Recall (%)	mAP (%)	mAP50-95 (%)	Time (ms/image)
**YOLOv5** [39]	75.3	68.2	70.6	**59.4**	0.8
**YOLOv5-P2BiP** [39]	77.6	63.3	69.3	56.8	0.76
**YOLOv6** [40]	70.5	61.6	66.3	52.1	**0.7**
**YOLOv7** [41]	55.7	55.6	54.9	40.8	1.2
**YOLOv8n** [42]	66.3	64.5	64.5	54	0.9
**YOLOv9** [43]	71.1	**68.5**	70.6	56.9	3.4
**YOLOv10** [44]	76.6	60.4	64.9	55.2	0.9
**YOLOv8-Ghost** [45]	**80.1**	64.5	69.9	57.3	1.5
**YOLOv8-P2** [46]	78	63.1	68.5	56.4	2.0
**YOLOv8-Ghost-P6** [47]	73.3	62.1	65.6	55.2	2.1
**YOLOv8-ALGP**	76.1	65.6	**71.3**	**59.4**	**0.7**

The experimental results are shown in Table 5, where YOLOv8-ALGP achieved the best performance in mAP and processing speed evaluation metrics. Especially in mAP, it is 0.7% and 6.8% higher than that of the second-best algorithm and the baseline algorithm YOLOv8 algorithm (Varghese et al. [42]). Compared to the other algorithms, the higher precision is the YOLOv8-Ghost (Zhang et al. [45]) and YOLOv8-P2 (Wang et al. [46]) algorithms, which are 80.1% and 78%, respectively. The better recalls are the YOLOv9 (Wang et al. [43]) and YOLOv5 (Feng et al. [39]) algorithms, both above 68%. However, a single high accuracy may lead to missed detections, and a single high recall may lead to false detections. mAP integrates accuracy and recall, and therefore focuses on the model’s mAP result metrics. Undoubtedly the best result in terms of mAP and mAP50-95 is the YOLOv8-ALGP algorithm. The best algorithms in terms of image processing speed are the YOLOv8-ALGP and YOLOv6 (Gupta et al. [40]) algorithms, yet the YOLOv6 performs poorly on other metrics.

Finally, YOLOv5-P2BiP (Feng et al. [39]), YOLOv7 (Wang et al. [41]), YOLOv10 (Wang et al. [44]), and YOLOv8-Ghost-P6 (Wang et al. [47]) algorithms are not ahead of the other algorithms in terms of metrics. By comprehensively analyzing the overall performance metrics of the various algorithms, our YOLOv8-ALGP achieved the best detection performance while maintaining the fast detection speed, which meets the defect detection requirements of industrial inspection.

Compared to other defect detection algorithms, further exploration is conducted. The automatic defect detection algorithm proposed by Mei et al. [48] performs better on defect-free samples, yet is not applicable to the defect dataset in this study compared to the YOLOv8-ALGP algorithm. The EDDs family of detectors proposed by Zhou et al. [49], designed for the characteristics of fabrics, relies more on low-level features, while aluminum alloys defect detection prioritizes high-level shape and edge features. He et al. [50] proposed a DDN system for steel plate defect detection using deep learning. It runs at 20 ft/s and has a mAP of 70% for the classification and detection tasks; in comparison, the mAP of YOLOv8-ALGP is 1.3% higher than that of the DDN system.

Comprehensively analyzing the performance metrics of various algorithms, YOLOv8-ALGP demonstrates the best performance in three key areas: fast image processing speed, mAP, and mAP50%–95%. This effectively meets the needs of defect detection in industrial production. Although this study focuses on common defect types, future work will involve improvements to the architecture to address a wider range of defect types that may occur in actual production.

## Conclusions

To address the problems of low accuracy and high cost of labor in the detection and classification of aluminum alloys defects by traditional methods, this work proposed a deep learning classification and detection method. The method can automatically learn defect feature representation without relying on manual feature extraction, achieving efficient and accurate defect detection and classification of aluminum alloys. The main research results of this paper are as follows.

(1) Aluminum alloys defect dataset is applied for training. Three common defect types are added to the dataset of 10 defects to expand the scope of detection classification. A mosaic data augmentation strategy is introduced to increase the diversity of the training dataset and improve the generalization ability of the model.

(2) An enhanced defect classification and detection network based on YOLOv8 is proposed for identifying aluminum alloys surface defects. Aiming at the characteristics of surface defects of aluminum profiles, the idea of light weight is added on the basis of YOLOv8 model. It shortens the training time, improves the detection accuracy and reduces the complexity of the model.

(3) The performance of the defect detection network is tested with 640 test datasets. Compared with YOLOv8, the improved YOLOv8-ALGP detection network has a higher mAP value of 71.3%. The average detection accuracy doubles for slender defects (such as jet), indicating that our proposed network model is effective in detection. In addition, we have listed some comparative graphs of the detection results of defect images to verify the effectiveness of the network model.

## Supporting information

Defeat_dataset (ZIP)
