# Supplementary figures and images for: Multi-defect detection and classification for aluminum alloys with enhanced YOLOv8
Source: PLoS One. 2025 Mar 20;20(3):e0316817. doi: 10.1371/journal.pone.0316817 (PMC11925294; doi:10.1371/journal.pone.0316817)

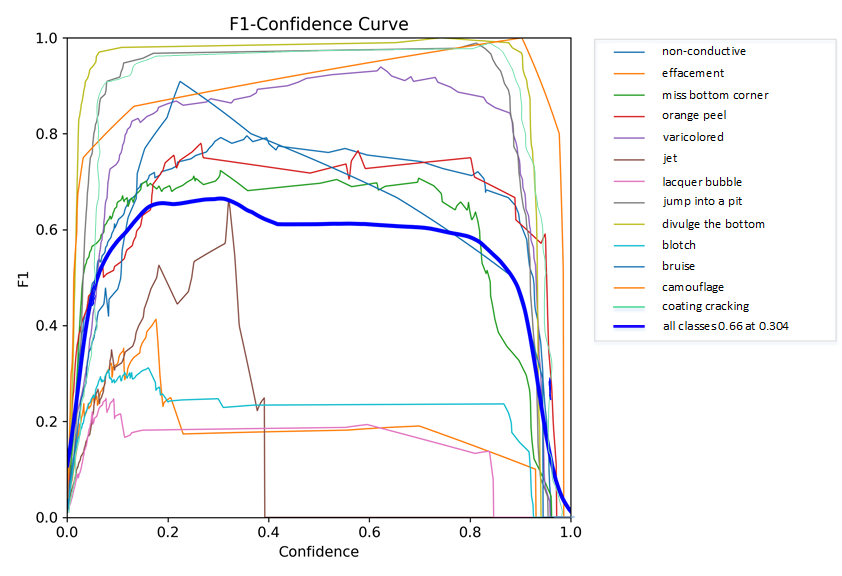

Supplement: Defeat_dataset [file pone.0316817.s001.zip › results_images/F1_curve.png]

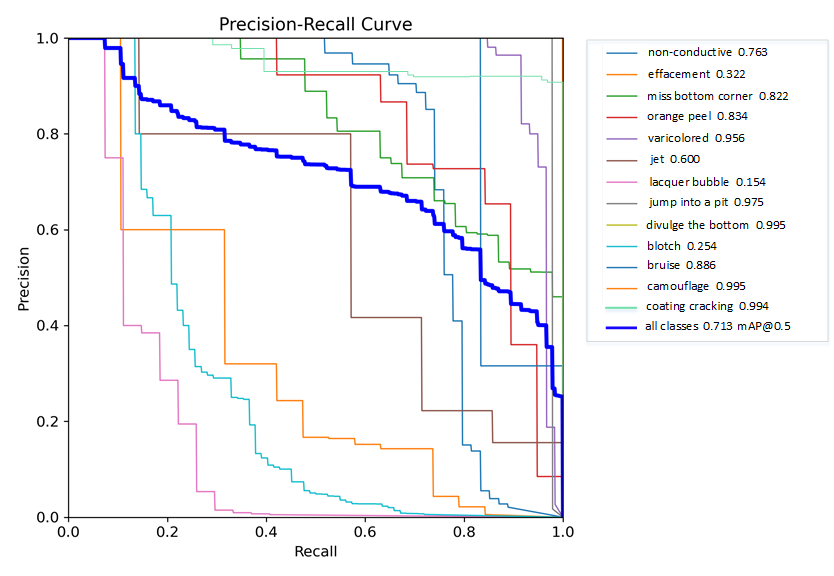

Supplement: Defeat_dataset [file pone.0316817.s001.zip › results_images/PR_curve.png]

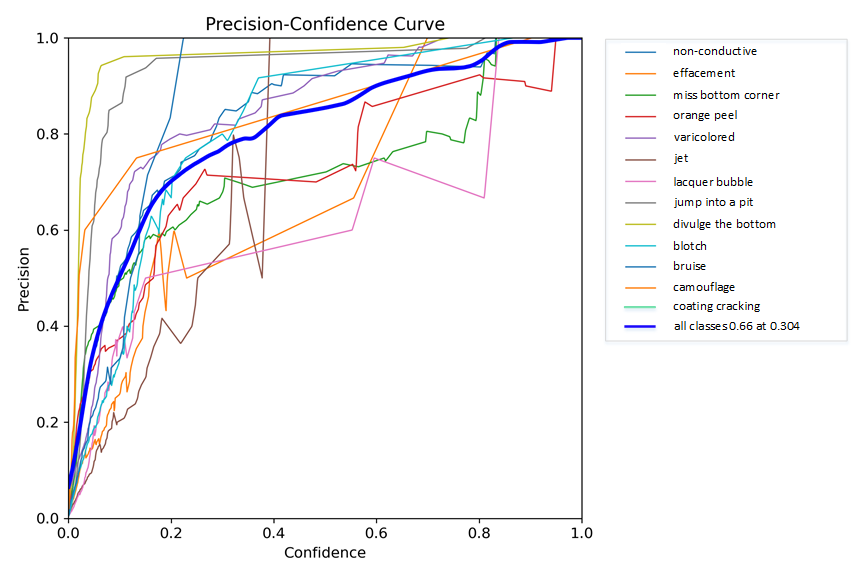

Supplement: Defeat_dataset [file pone.0316817.s001.zip › results_images/P_curve.png]

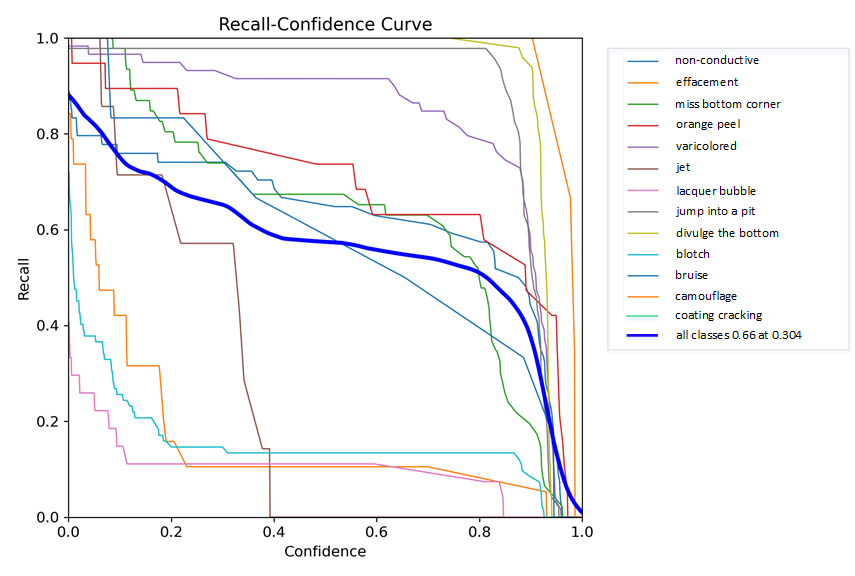

Supplement: Defeat_dataset [file pone.0316817.s001.zip › results_images/R_curve.png]

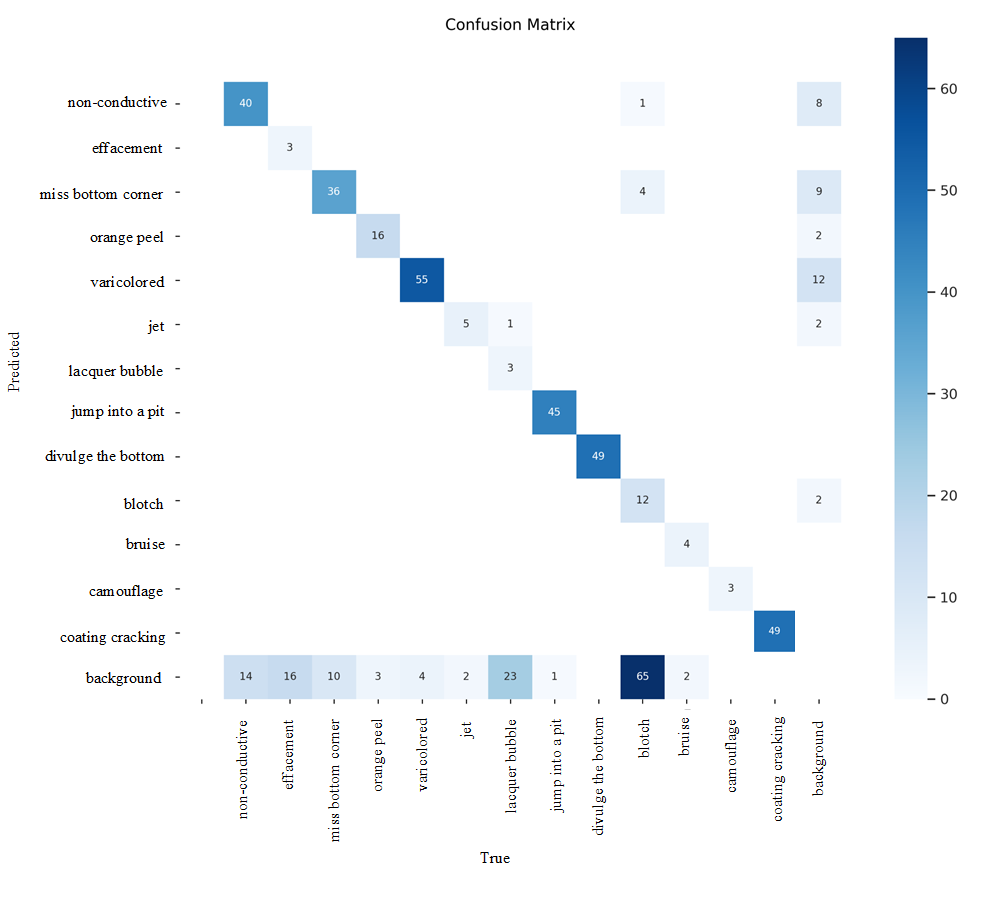

Supplement: Defeat_dataset [file pone.0316817.s001.zip › results_images/confusion_matrix.png]

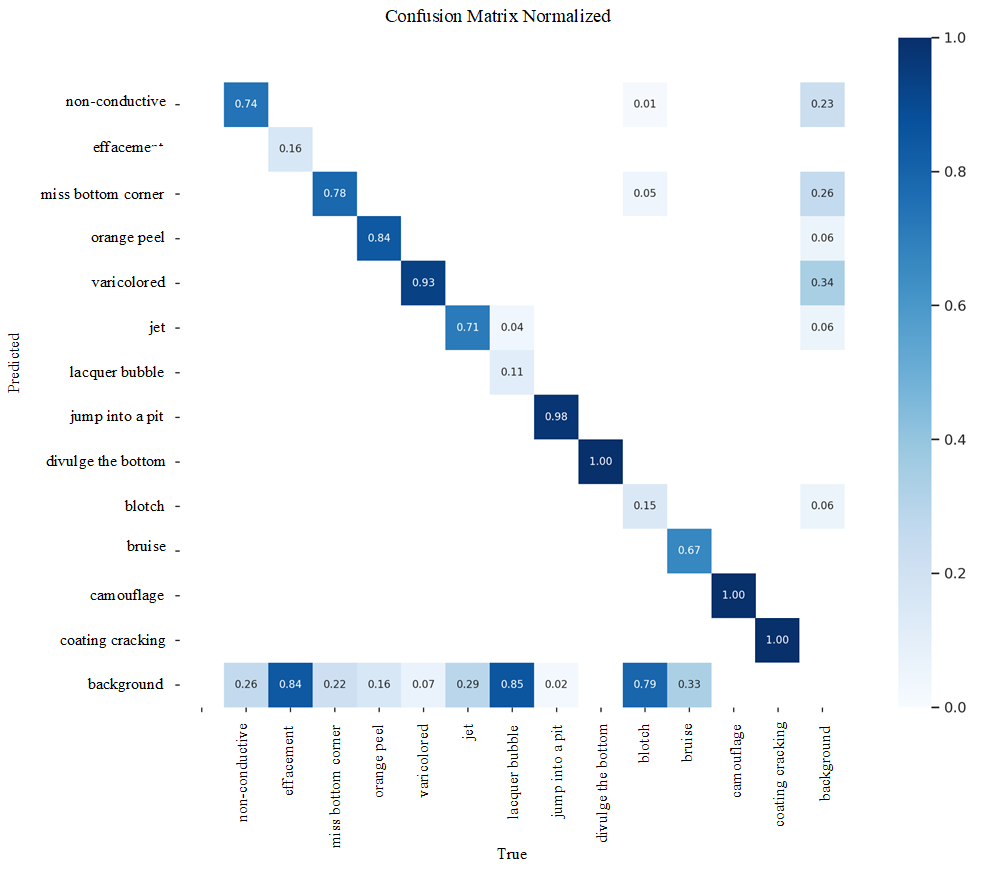

Supplement: Defeat_dataset [file pone.0316817.s001.zip › results_images/confusion_matrix_normalized.png]

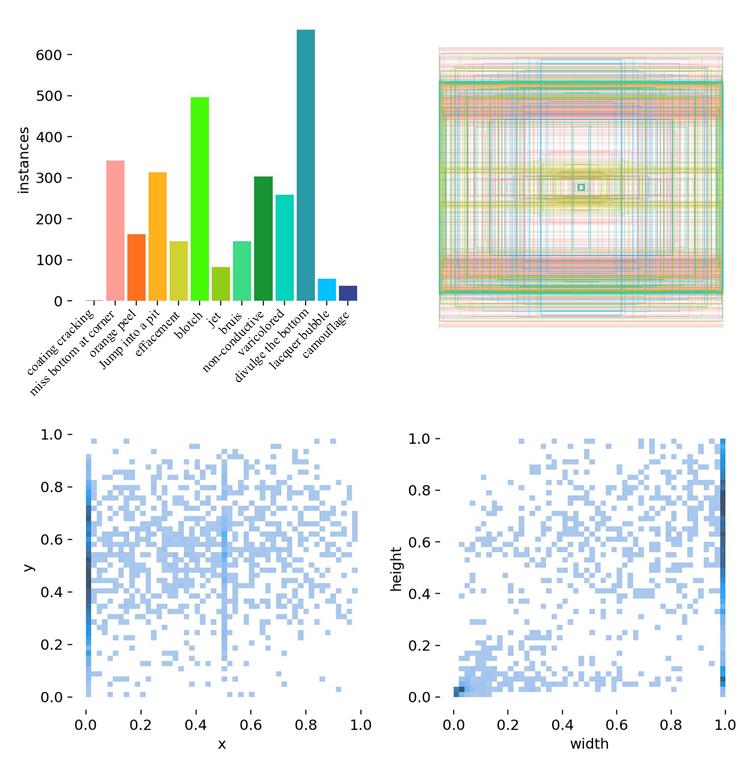

Supplement: Defeat_dataset [file pone.0316817.s001.zip › results_images/labels.jpg]

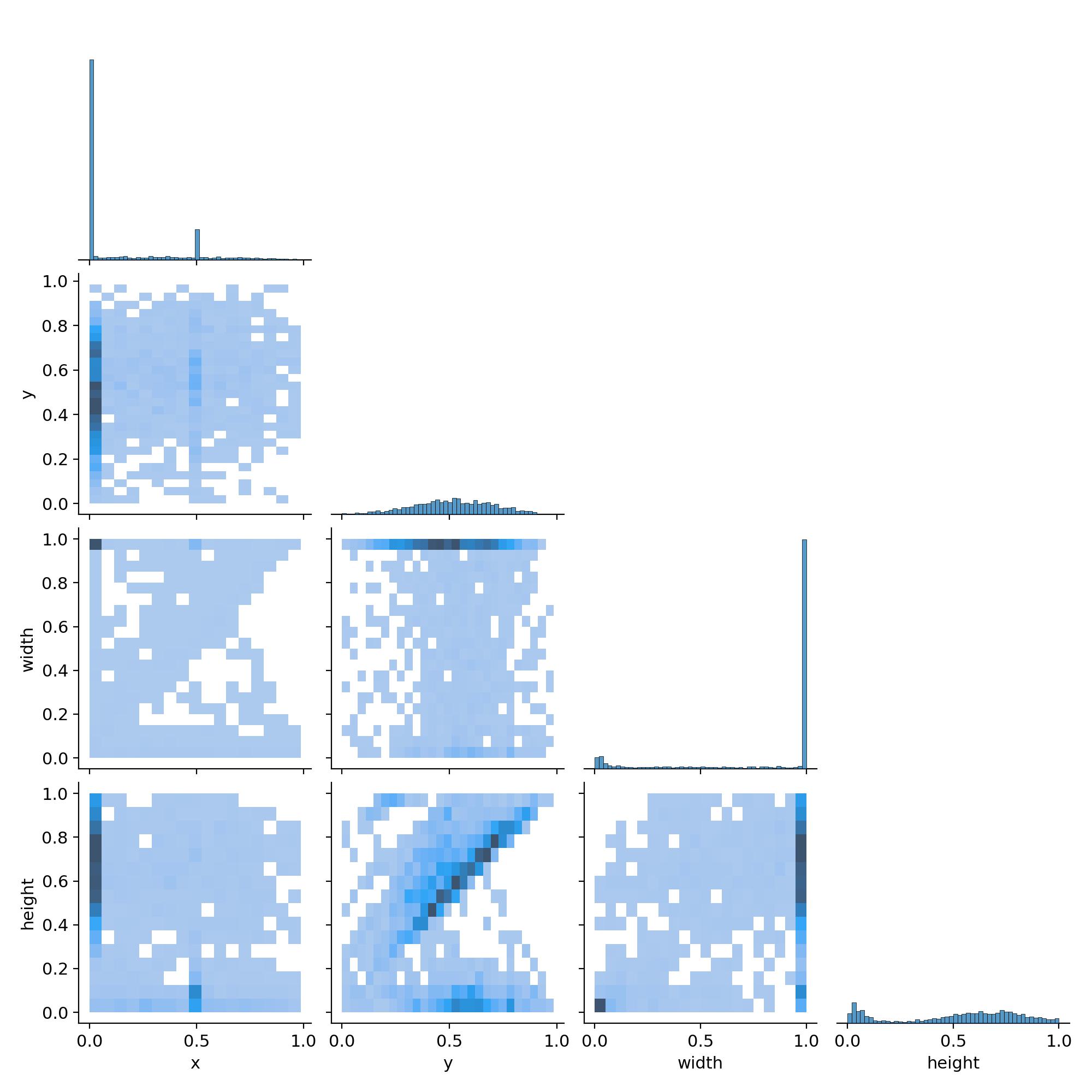

Supplement: Defeat_dataset [file pone.0316817.s001.zip › results_images/labels_correlogram.jpg]

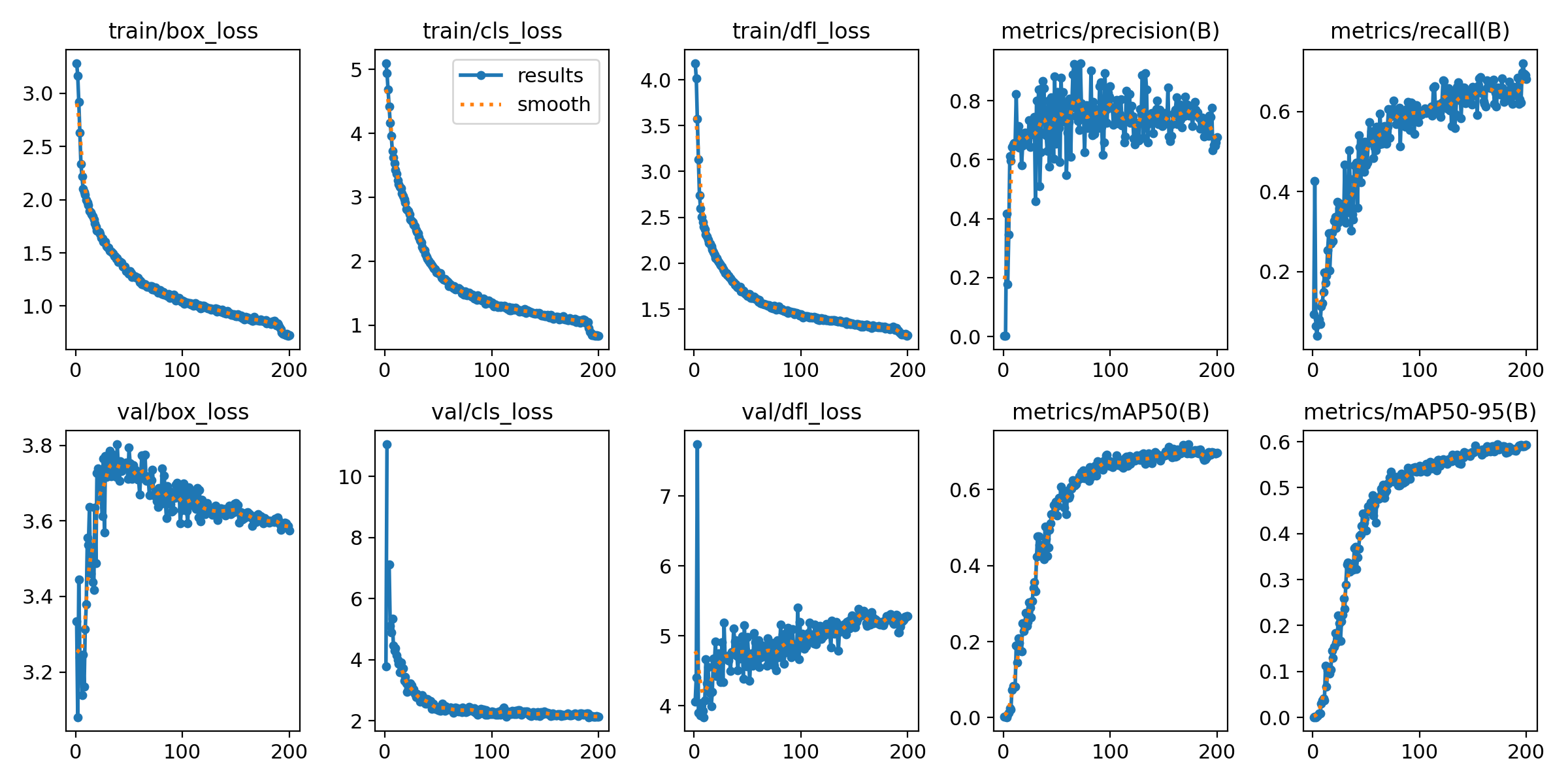

Supplement: Defeat_dataset [file pone.0316817.s001.zip › results_images/results.png]

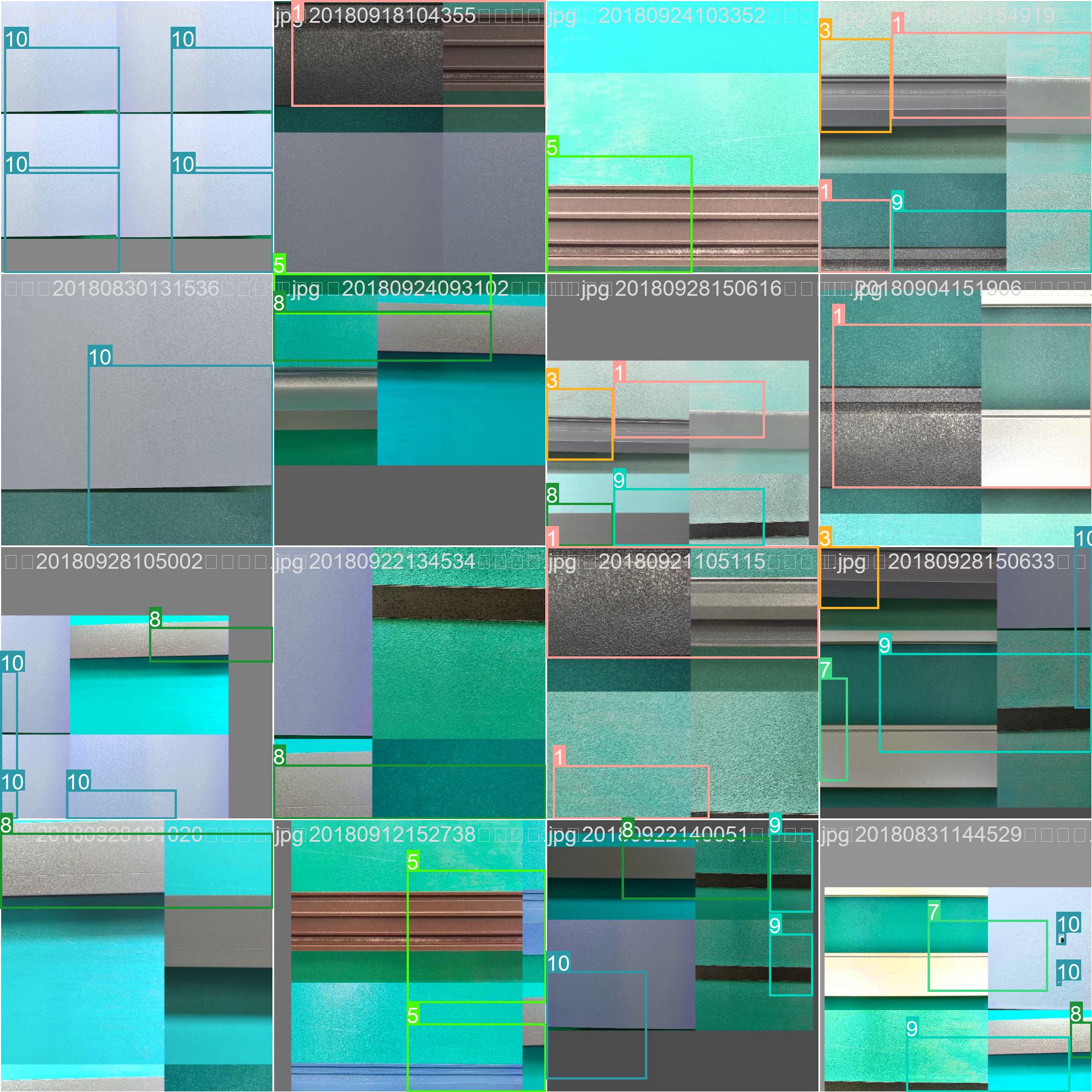

Supplement: Defeat_dataset [file pone.0316817.s001.zip › results_images/train_batch0.jpg]

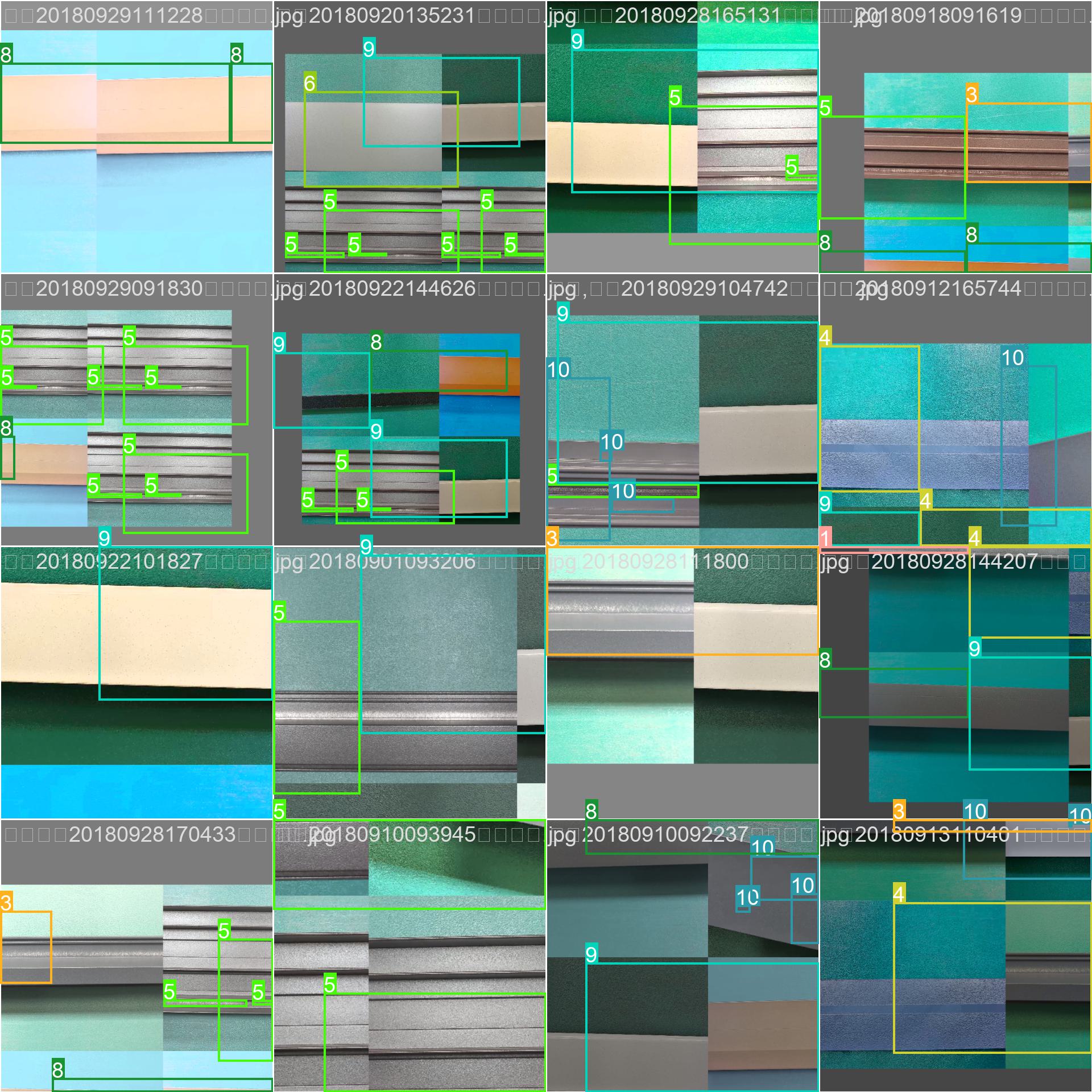

Supplement: Defeat_dataset [file pone.0316817.s001.zip › results_images/train_batch1.jpg]

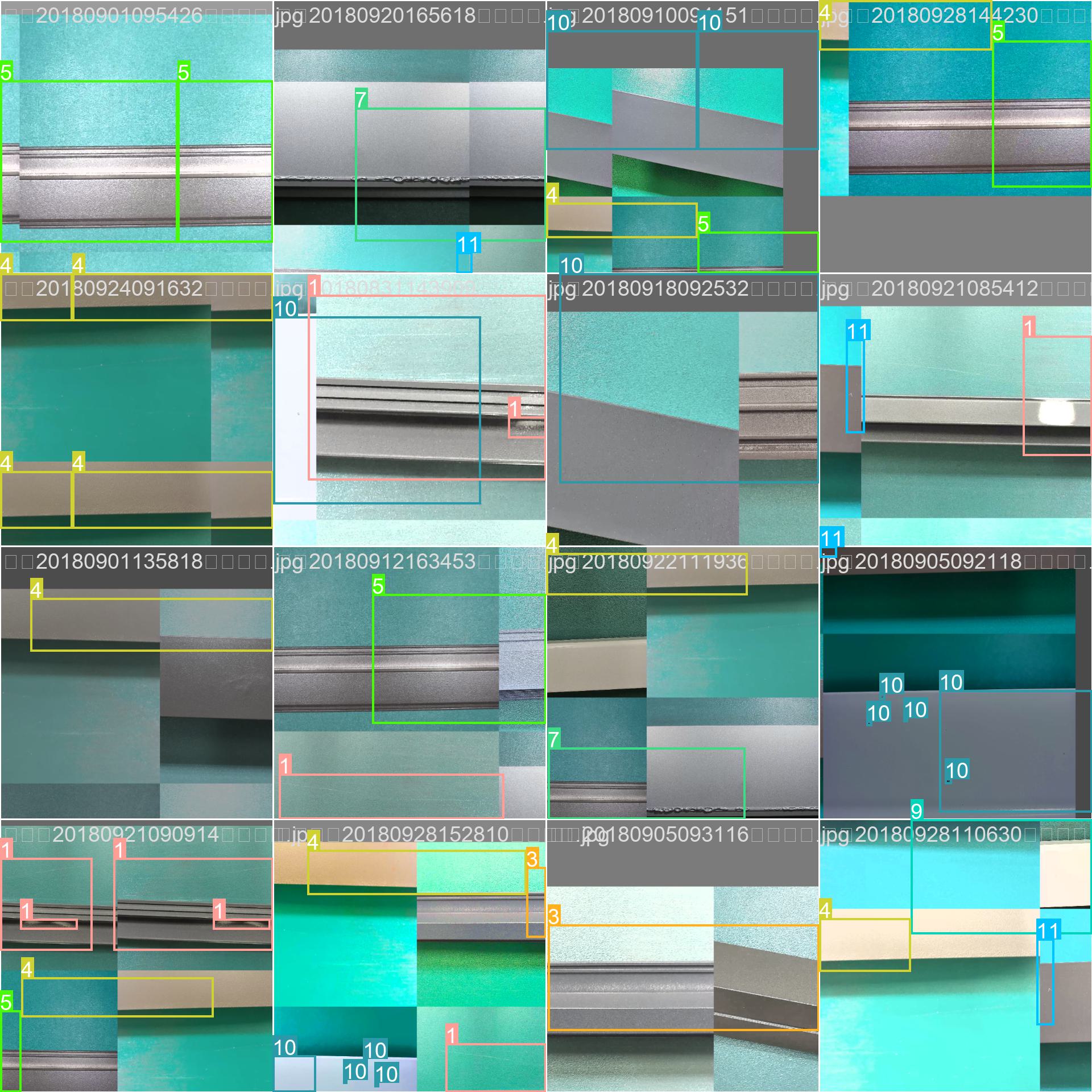

Supplement: Defeat_dataset [file pone.0316817.s001.zip › results_images/train_batch2.jpg]

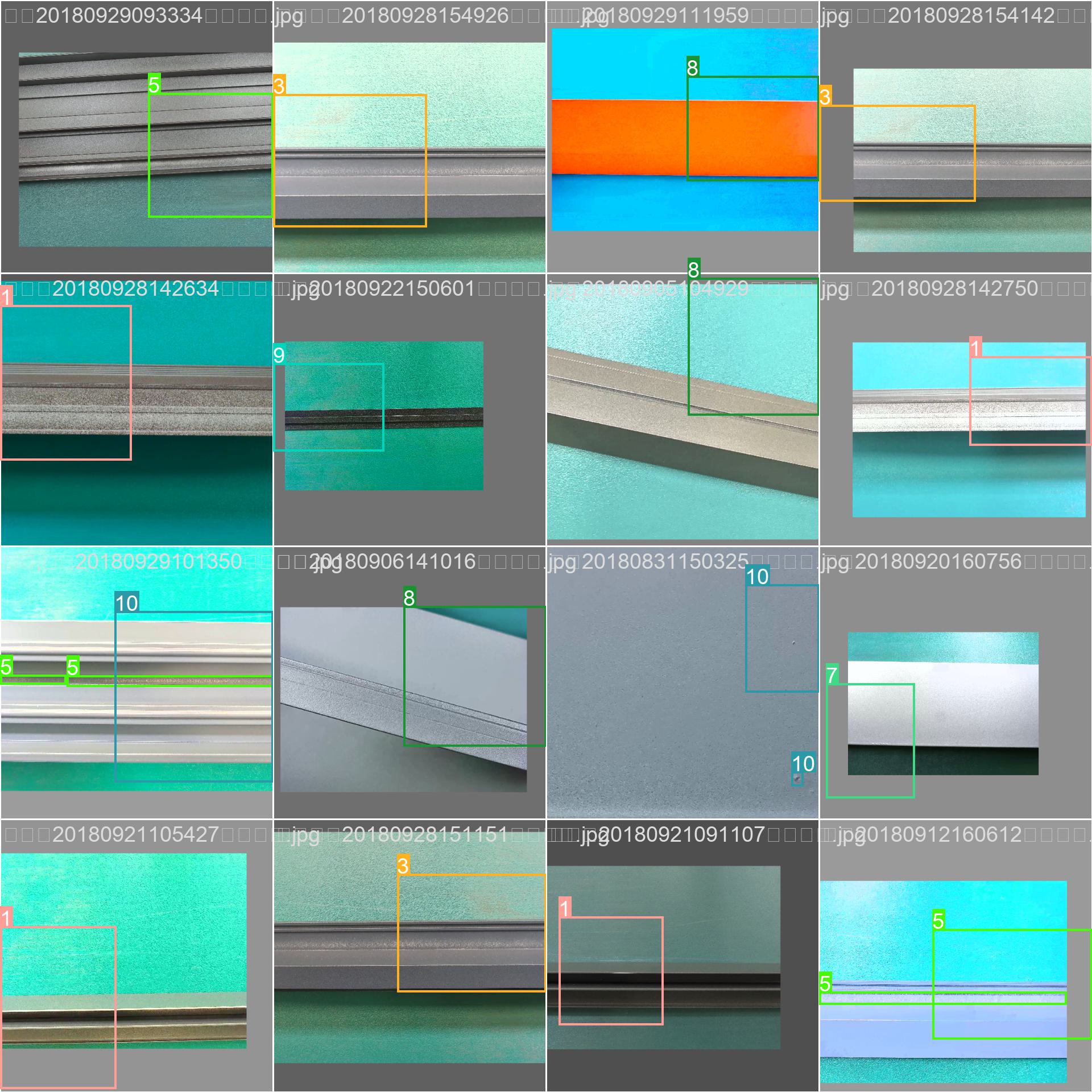

Supplement: Defeat_dataset [file pone.0316817.s001.zip › results_images/train_batch26980.jpg]

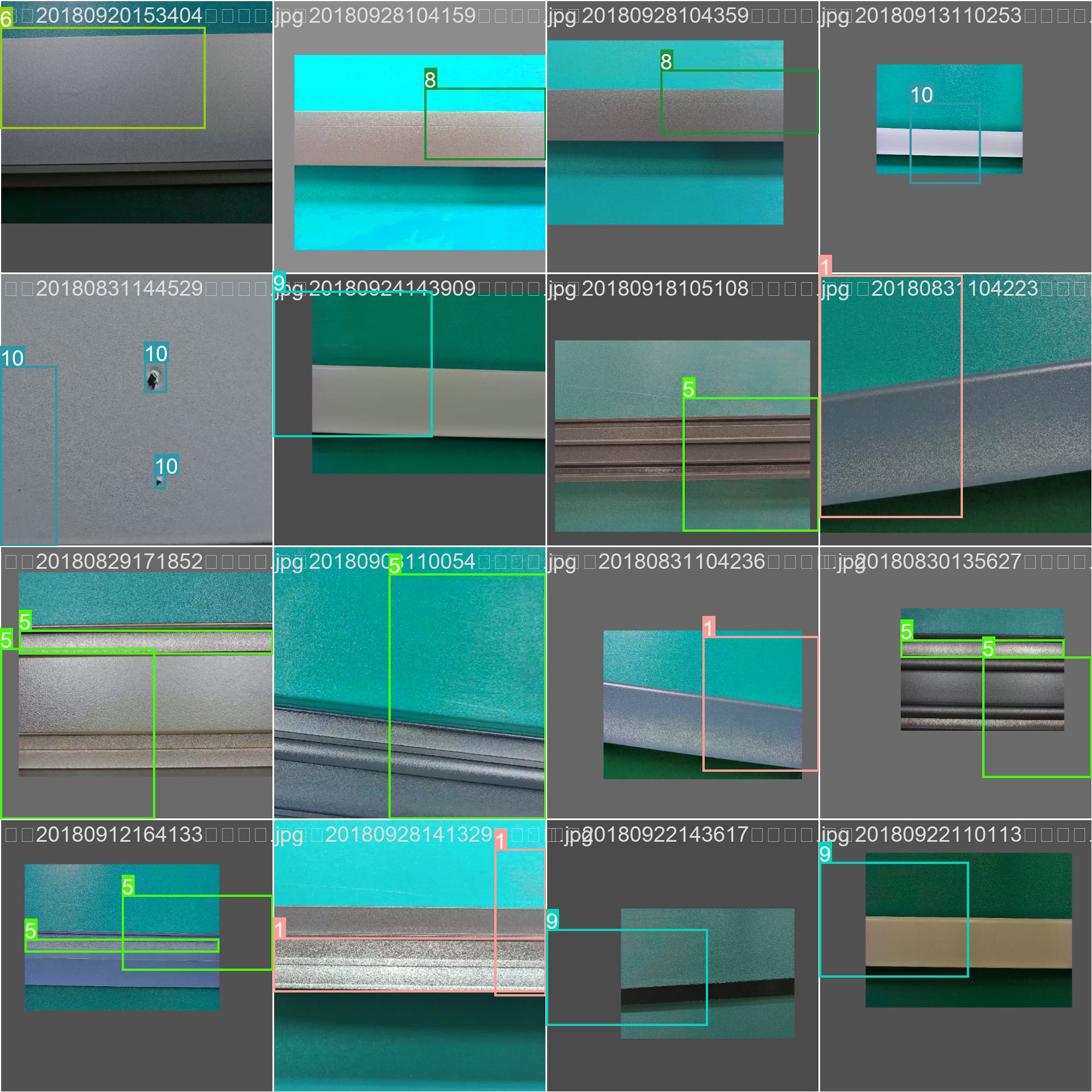

Supplement: Defeat_dataset [file pone.0316817.s001.zip › results_images/train_batch26981.jpg]

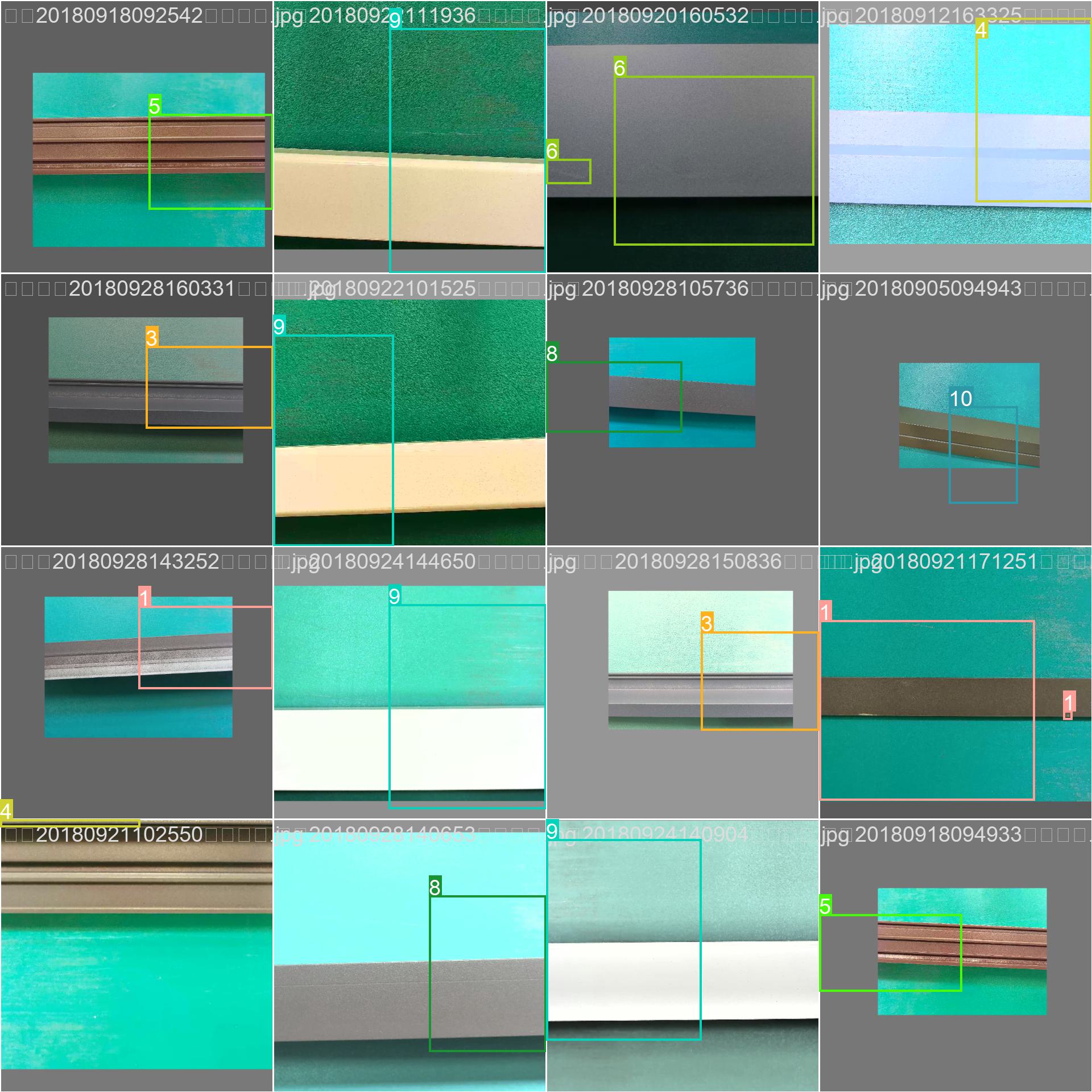

Supplement: Defeat_dataset [file pone.0316817.s001.zip › results_images/train_batch26982.jpg]

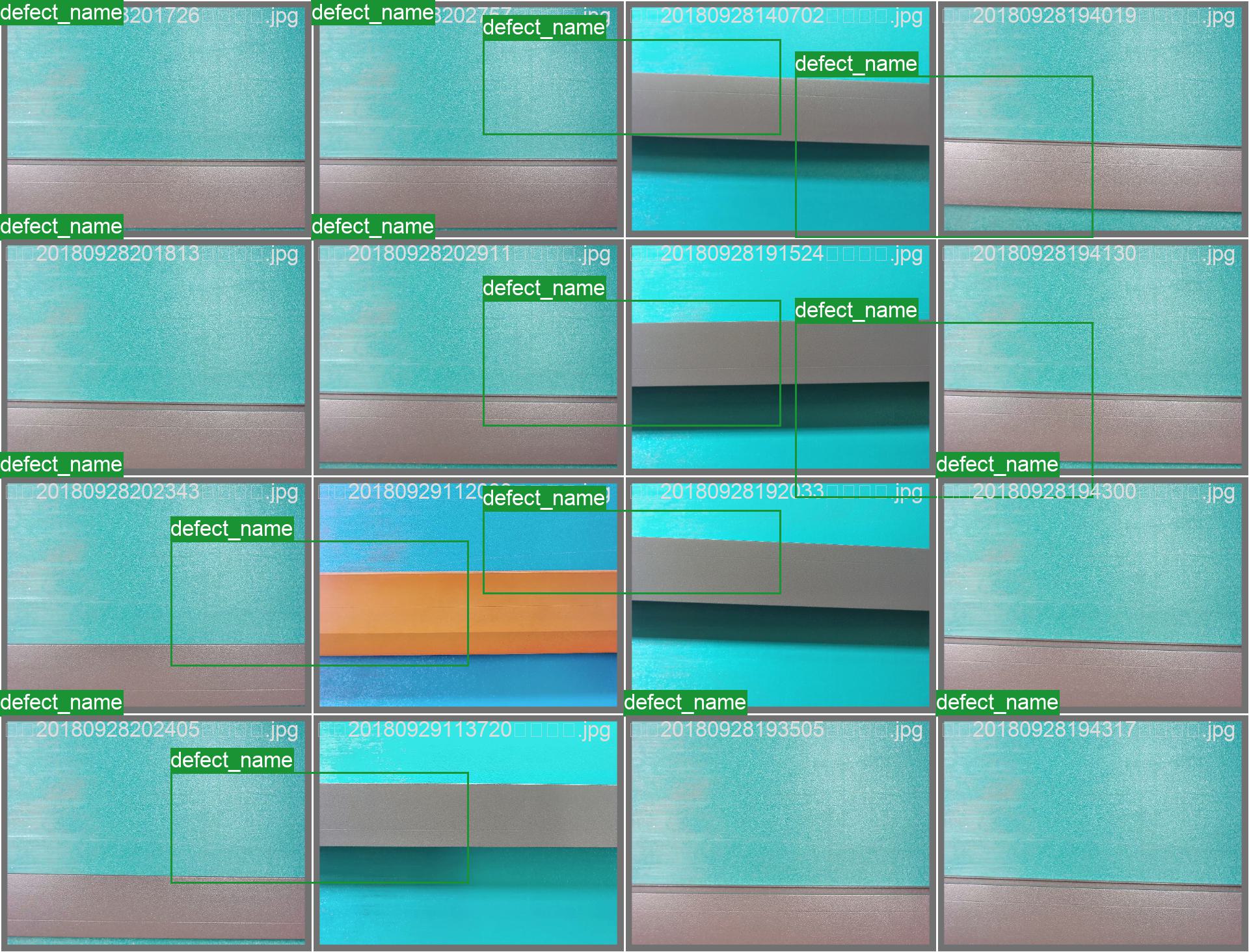

Supplement: Defeat_dataset [file pone.0316817.s001.zip › results_images/val_batch0_labels.jpg]
